# A Novel Model on DST-Induced Transplantation Tolerance by the Transfer of Self-Specific Donor tTregs to a Haplotype-Matched Organ Recipient

**DOI:** 10.3389/fimmu.2017.00009

**Published:** 2017-02-21

**Authors:** Angelica Maria Mohr Gregoriussen, Henrik Georg Bohr

**Affiliations:** ^1^Center for the Philosophy of Nature and Science Studies (CPNSS), University of Copenhagen, Copenhagen, Denmark; ^2^Department of Chemistry, The Technical University of Denmark, Lyngby, Denmark

**Keywords:** Tregs, indirect alloantigen presentation, direct alloantigen presentation, MHC-II recruitment, DST, transplantation tolerance, haplotype-matched, self-tolerance

## Abstract

Donor-specific blood transfusion (DST) can lead to significant prolongation of allograft survival in experimental animal models and sometimes human recipients of solid organs. The mechanisms responsible for the beneficial effect on graft survival have been a topic of research and debate for decades and are not yet fully elucidated. Once we discover how the details of the mechanisms involved are linked, we could be within reach of a procedure making it possible to establish donor-specific tolerance with minimal or no immunosuppressive medication. Today, it is well established that CD4+Foxp3+ regulatory T cells (Tregs) are indispensable for maintaining immunological self-tolerance. A large number of animal studies have also shown that Tregs are essential for establishing and maintaining transplantation tolerance. In this paper, we present a hypothesis of one H2-haplotype-matched DST-induced transplantation tolerance (in mice). The formulated hypothesis is based on a re-interpretation of data from an immunogenetic experiment published by Niimi and colleagues in 2000. It is of importance that the naïve recipient mice in this study were never immunosuppressed and were therefore fully immune competent during the course of tolerance induction. Based on the immunological status of the recipients, we suggest that one H2-haplotype-matched self-specific Tregs derived from the transfusion blood can be activated and multiply in the host by binding to antigen-presenting cells presenting allopeptides in their major histocompatibility complex (MHC) class II (MHC-II). We also suggest that the endothelial and epithelial cells within the solid organ allograft upregulate the expression of MHC-II and attract the expanded Treg population to suppress inflammation within the graft. We further suggest that this biological process, here termed MHC-II recruitment, is a vital survival mechanism for organs (or the organism in general) when attacked by an immune system.

## Introduction

Today, transplantation tolerance in humans who receive an organ from a genetically disparate donor can very rarely be achieved without life-long administration of immunosuppressive therapy. While the current, non-specific immunosuppressive drugs are very good at inducing short-term graft survival, the impact on long-term engraftment has remained unchanged because most of the patients lose their transplants due to chronic rejection. Furthermore, due to general suppression of the immune system, the patients are exposed to a higher risk of infections and developing cancer. Therefore, the ultimate goal is to find a way to induce permanent donor-specific tolerance (1, 2).

Long-term alloantigen-specific transplantation tolerance of solid organs can easily be induced in rodents without continuous supply of immunosuppressants (3). Although the many successful approaches in experimental animal models have failed when attempted in humans (4, 5), there are cases of drug-free humans carrying well-tolerated organs, such as kidneys and livers, from genetically disparate donors (6, 7). These rare cases of spontaneous tolerance (also termed operational tolerance) to a foreign organ provide hope that transplantation tolerance with no or minimal immune-suppressive medication can be achieved in humans.

A widely used strategy to induce long-term transplantation tolerance in experimental animal models is to introduce the organ recipient to alloantigens in the form of blood transfusion before transplantation (8). Multiple blood transfusions before transplantation have also been shown to prolong kidney graft survival in humans and became a widely used strategy in the 1970s (9–11). However, the procedure may also sensitize the recipient to donor antigens. Therefore, following the development of highly efficient immune suppressive drugs in the 1980s, deliberate pretransplant blood transfusion has been either abolished or abandoned in virtually every hospital worldwide (12).

Sharing of a HLA/H2 haplotype between the recipient and blood donor in both humans and experimental animal models is associated with prolongation of graft survival. The effect is further optimized when the haplotype shared blood is donor specific, i.e., taken from the organ donor [donor-specific blood transfusion (DST)] (13–15).

The mechanisms responsible for DST-induced unresponsiveness are not fully understood. However, many experimental studies in animals indicate that perhaps the most powerful mechanism involved in prolongation of allograft survival is the suppressor function of Foxp3 CD4+CD25+ regulatory T cells (Tregs). Tregs are also found to be indispensable for maintaining peripheral self-tolerance. How DST may induce Tregs has been intensively studied for decades.

One central issue is whether donor-derived leukocytes are actively involved in inducing transplantation tolerance, and if so, how and to what extent. In this paper, we re-interpret an immunogenetic experiment conducted 17 years ago by Niimi et al., and we present a hypothesis that suggests that actively involved donor blood-derived Tregs and the donor organ itself are crucial for the prolongation of the allograft survival achieved in their study.

## Current Hypothesis on Alloantigen-Induced Transplantation Tolerance

Donor cells from variable immune lineages may enter the recipient *via* the donor organ as “passenger cells” and *pretransplanted donor blood*. In the early 1990s, Thomas E. Starzl and his colleagues proposed a model of microchimerism and transplantation tolerance. According to this model, allograft acceptance is the effect of a balanced double immune reaction between donor and recipient cells, which is comparable to a mutual graft-versus-host/host-versus-graft reaction, and causes reciprocal clonal exhaustion. Although the reaction eventually attenuates, it remains at a low level and keeps a dangerous increase of either of the two reactions in check. The persistence of microchimeric cells is therefore considered to be a prerequisite for long-term allograft survival in this model (16).

In support of this hypothesis, donor leukocytes in the recycling lymphoid pool of the host have been identified as important for inducing response reduction in experimental studies with animals (17).

Hypotheses that consider donor leukocytes as active participants in tolerance induction have been challenged by other studies showing, for example, that reduced immunogenicity can be obtained if passenger cells are removed from the transplant (18). Additionally, there is strong evidence that the pathway of indirectly presented allopeptides [i.e., allopeptides that are presented by host antigen-presenting cells (APCs)] is used by Tregs for immune regulation (19–21). Microchimeric cells are therefore assumed to function mainly as a depot for alloantigens for the recipient immune system (20).

Data from non-immunosuppressed human recipients of one HLA-DR-shared pretransplant blood transfusion has spawned one of the latest models on the subject.

The data showed that these recipients had a reduction in acute rejection episodes compared to those patients who also received antithymocyte globulin as induction therapy (22). In accordance with current understanding, it is suggested that Tregs and effector T cells may be responsible for a substantial part of the unresponsiveness toward the donor organ, and further that allospecific effector T cells pick up allopeptides from the transplanted organ and present them in their HLA class II molecules. The HLA class II/allopeptide complex may then serve as a specific target for Tregs induced by the donor blood. It is further suggested that when binding to the effector T-cells, Tregs cause cell lysis. This hypothesis is supported by a study of *in vitro*-generated CD4+CD25+ T cell clones that showed that these cells lysed autologous cytotoxic T cells as long as the proper allopeptide was presented in the context of self-major histocompatibility complex class II (MHC-II) (23). Recognition of MHC/allopeptide complexes by the blood transfusion-induced Tregs presupposes that blood and organ donor share one or more alloantigens and that these shared alloantigens are mismatched to the recipient.

Unfortunately, this model does not explain allotolerance in mice because mice T-cells do not produce MHC-II. Therefore, a different model is required to explain similar phenomena that occur in mice.

## Features of Tregs Which are Relevant to Our Hypothesis

The discovery by Sakaguchi et al. in 1995 that a subset within the CD4+ T cell population that expresses CD25 are responsible for maintaining immunological self-tolerance in mice (24) triggered enormous research activity on the subject. Subsequently, attention was further focused on this T-cell subset with the discovery of Foxp3 (forkhead box P3), a transcription factor that has proven to be a driving force for the development of Tregs in both mice and humans. Today, it is well established that Tregs are specialized for immune suppression of T-cell-induced autoimmune responses. It is also well established that Tregs play a fundamental role in immune suppression of responses toward non-self-antigens such as allografts in many different animal models. The role of Tregs in immune tolerance has been extensively reviewed elsewhere (5, 25). The majority of Tregs that populate the peripheral lymphocyte pool develop in the thymus (tTregs or nTregs). Unlike conventional T cells, tTregs are positively selected by recognition of self-antigens, including peripheral tissue-specific antigenic peptides driven by AIRE on medullary thymic epithelial cells (26, 27).

Since tTregs are biased toward self-antigens, they are interpreted as crucial for maintaining peripheral immunological self-tolerance (28). Tregs may also be induced in the periphery (pTregs) or generated *in vitro* (iTregs) from the naïve CD4+ T cells population when encountering MHC/peptide complexes on dendritic cells in tolerogenic environments, such as in the presence of TGF-β and interleukine-2 (IL-2) (29–34); i/p Tregs have been extensively reviewed in Ref. (35). In healthy individuals, pTregs are mainly located in mucosal surfaces such as in the gut and lungs where they control local inflammation (36). Tregs that recognize non-self-antigens are assumed to hold real promise for inducing specific tolerance to foreign graft antigens (28, 37).

Regulatory T cells may lose their suppressive activity or take on an effector phenotype. The longevity and stability of Tregs are associated with high expression of Foxp3. Foxp3 promotes the expression T-cell activation markers such as CTLA-4 and CD25, while it may inhibit the expression of IL-2 (38, 39). The stability of Foxp3 expression can be determined by the demethylation status of a conserved GpG-rich intronic region within the *foxp3*-locus upstream of exon-1, also termed TSDR (40). For tTregs, the expression of Foxp3 is already initiated during early Treg development as Tregs pass through thymic medulla. Although Tregs that leave the thymus may not display a fully demethylated TSDR, experimental studies in mice suggest that commitment to a stable Treg lineage is established during early thymic development (5, 41). It has also been shown that a substantial portion of tTregs that leave the thymus are in an antigen-primed stage and ready to regulate (25, 42).

Animal studies have shown that the intrathymic expression of Foxp3 and the further development of nTreg in the periphery as well as maintenance of the Treg linage are dependent on antigen-specific TCR signaling (43–45). Since tTregs constantly meet self-antigens in the periphery, they are considered as a more stable Treg line than pTregs which will lack sufficient TCR stimulus once the foreign antigen is eliminated (5).

Studies on CD62L+ (naïve) Tregs show that they proliferate vigorously *in vivo* upon antigen-specific stimulation and in the presence of IL-2, and die after exerting their suppressive activity. The life cycle of Tregs is interpreted as crucial for maintaining immune homeostasis (25, 46, 47). An increasing number of studies in humans and mice suggest that Tregs are distributed throughout non-lymphoid tissues where they are active in suppression of inflammatory responses (33, 48). It has also been demonstrated that memory-like Treg take up residence in the target tissue after resolution of an inflammatory response. Moreover, these Tregs were primed to suppress a subsequent autoimmune response when the self-antigen was re-expressed by the inflamed tissue (49).

In line with this discovery, Tregs have been isolated from tolerated skin allografts in mice (50). *In vitro* studies have demonstrated that Tregs can regulate immune responses on multiple levels and *via* several different mechanisms. For example, they can inhibit activation and function of APCs and produce immunoregulatory cytokines, such as TGF-β and IL-10, which act directly on effector T cells by impeding their priming and effector function (51). Tregs can also cause cytotoxic T-cell death by FasL/fas-mediated apoptosis (52). The regulatory mechanisms of Tregs have been reviewed by Povoleri et al. with references to the original literature (32). Another important suppressor function of Tregs is the ability to cause “linked” or “bystander” suppression of a population of effector T cells with specificity for a different antigen and to render these immunocompetent T cells tolerant over time. Furthermore, these tolerant cells might again confer tolerance to T cells that recognize a third-party antigen. This phenomenon is termed “infectious tolerance.” The chain reaction of tolerance induction requires that Tregs and T cells as well as the various alloantigenic peptides are attached to the same APC (13, 53, 54). The phenomena of linked suppression and infectious tolerance have been reviewed by Lechler et al. (55).

Although antigen-specific signaling *via* TCRs is important for Treg priming and function, recent studies in mice have shown that Tregs (that can regulate both direct and indirect presentation pathways) are needed to induce indefinite transplantation tolerance (29, 56). This clearly suggests that prevention of chronic rejection favors a persistent homogeneity between the direct and indirect pathway of alloantigen presentation/recognition, and example being that the recipient and donor share a HLA-DR/MHC-class II molecule or a haplotype.

### Phenomena of Allograft Survival from an Immunogenetic Study by Niimi et al.

Much of our understanding of the mechanisms involved in induced transplantation tolerance is derived from studies in mice. We know that there are significant differences between the immune system of a naïve, often genetically and immunologically altered, mouse model and an adult human being, who has been exposed to several infections and vaccines throughout life. Yet, these studies are a rich source of ideas about some fundamental principles of immune regulation and how the mechanisms involved may be manipulated/adjusted according to requirements (13). There are two main reasons why we have chosen to analyze the phenomena that occur in this study. (1) Through its examination of the impact of organ donor MHC type and its relationship to blood donor and recipient MHC type on prolongation of allograft survival, this study takes into account all three parties involved—the recipient, the blood donor, and the organ donor. (2) Unlike the majority of experiments on tolerance, the recipients in this study were at no time immune suppressed. Thus, the immune status of the recipients was “active” at the time of transfusion and during the initial stage of tolerance induction. Therefore, interference of artificially forced immune phenomena such as antibody-induced clonal anergy can be ruled out. On the other hand, this minimally manipulated experiment may allow other mechanisms to become more apparent.

**Figure 1:**
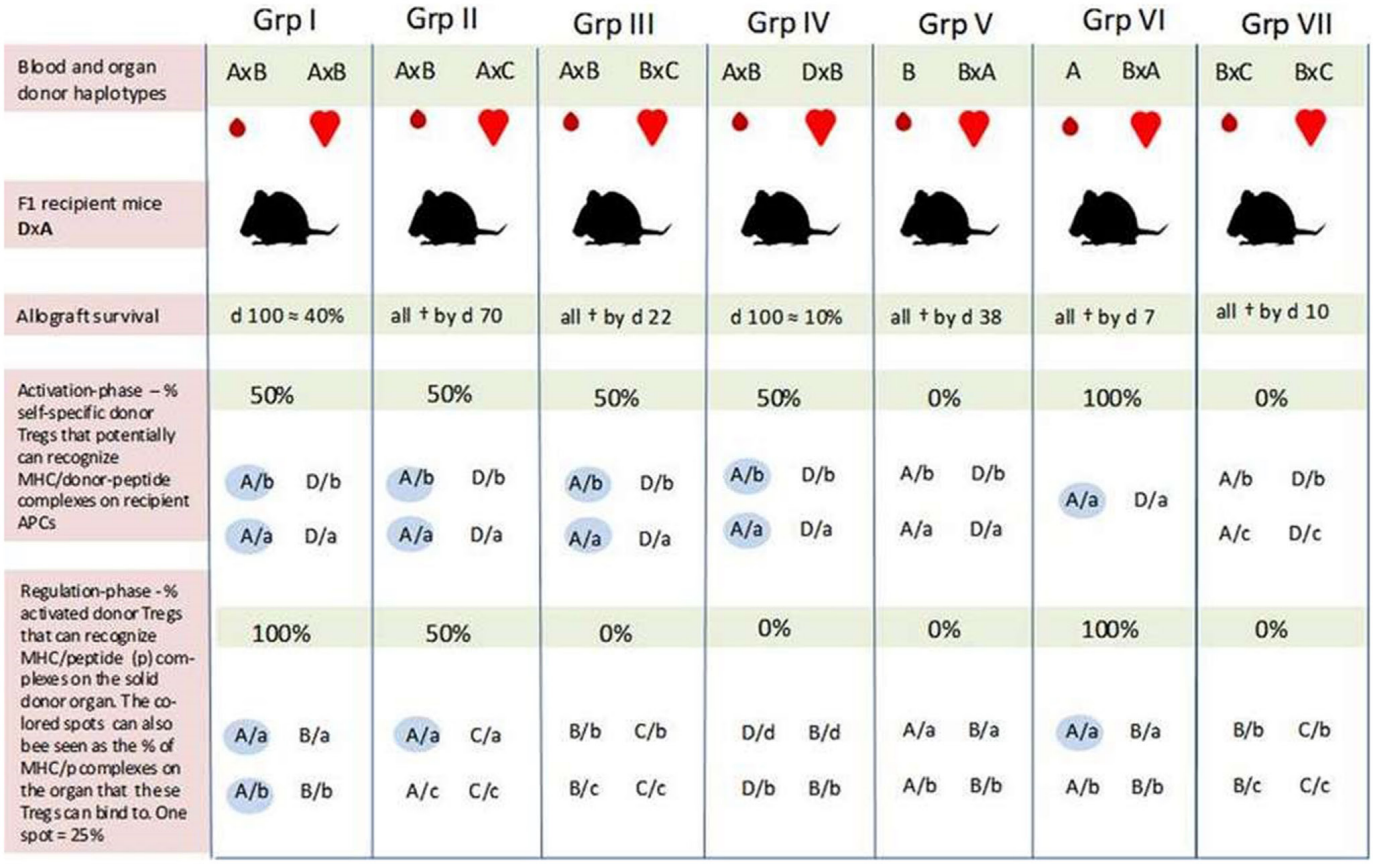
**An overview of self-major histocompatibility complex class II (MHC-II)/peptide share between the recipient and blood donor and the possibility for induction of regulatory T cells (Tregs) which can recognize the same peptide/MHC complex on host and donor antigen-presenting cells (APCs) in the study by Niimi et al**. All F1 recipient mice (DxA) were transfused with matched/mismatched blood prior to transplantation. One week later, the recipients were transplanted with one haplotype-matched or fully mismatched hearts. Allograft survival was optimal in recipients of one haplotype-matched donor-specific blood transfusion (DST) (Grp I), while the graft was rejected immediately in recipients transfused with fully mismatched DST (in Grp VII). In Grp I, there is the potential for activation of Tregs which can bind to 50% of the MHC-II/allopeptide complexes on the donor organ (AxB) (blue spots in the bottom row). Generation of Tregs with similar binding characteristics may also occur in Grp IV and V; only in these two cases, the donor-Tregs will either not recognize MHC/peptide complexes on the donor organ (Grp IV) or the recipient APC (Grp V). The 10% graft survival by day 100 was achieved in Grp IV, while all hearts had been rejected by day 38 in Grp V. Prolongation of graft survival achieved in Grp IV and V, and in particular the different outcomes in these two groups indicate that the indirect pathway of alloantigen presentation plays a pivotal role in tolerance induction—i.e., that the mismatched peptide (b) must be presented by host APC (A or D). How can the different outcomes then be explained? Passenger cells such as donor DCs have mainly been associated with acute rejection episodes. However, the more successful outcome of graft survival in Grp I seems to be linked to the blood donor after all. As suggested by our hypothesis, blood donor-derived self-specific tTregs may be responsible. These hypothetical tTregs can bind to both pathways of allo-antigen presentation (e.g., peptide b presented by MHC-A on host and donor APCs) and may succeed in disarming the initiation of a deleterious alloresponse. This tolerogenic situation may again create favorable conditions for the formation of a more solid network consisting of several tolerance-promoting mechanisms. Self-specific tTregs derived from H2/haplotype-matched donor blood may also explain the different outcomes in Grp IV and V by inducing linked suppression and infectious tolerance against the graft *via* host APCs (Grp IV). Prolongation of graft survival in Grp II may be due to some allopeptides derived from the “b” and “c” genetic backgrounds being identical. Furthermore, self-specific (A/a) donor and recipient tTregs may also contribute to the tolerance achieved in this group. As the results in Grp VI indicate, these hypothetical tTregs are not sufficient to induce tolerance on their own in this experiment setting. One explanation may be that there is a need for an inflammatory condition to activate the regulatory system. All values are our own estimation based on a graphical presentation in the article of Niimi et al. F1 combinations: A, C57BL/10; B, BALB/c; C, SJL; D, CBA, “a, b, c, and d” represent self-peptides derived from the inbred strains designated as A, B, C, and D, respectively. Grp, group; †, allograft rejection; 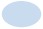, self-MHC/peptide complexes on host APC or the solid donor organ that may be recognized by self-specific donor Tregs.

In the Niimi experiment, recipient (F1) mice were transfused with one haplotype-matched or fully mismatched donor blood 7 days before transplantation. The recipient mice were then transplanted with a heart of different F1 mice with which they shared one haplotype (groups I, II, IV, V, and VI) or none (groups III and VII). All combinations are illustrated in Figure 1.

Two sets of experiments with different blood donors were conducted to ensure that the effects of pretransplant blood transfusion were not restricted to a specific strain combination. The results were similar.

Optimal prolongation of graft survival was achieved in recipient mice which received one haplotype-matched DST (approximately 40% at day 100) (I). Graft survival at day 100 was also obtained in 10% of the recipients that shared one haplotype with the blood donor and the mismatched haplotype with the organ donor (IV). The survival curve in this group was quite similar to that in which the recipient and both donors shared the same haplotype but were mismatched for the other haplotype (II). However, in the latter test group, all hearts had been rejected by day 70. Recipients transfused with homozygous blood from a mismatched donor obtained moderate graft prolongation (all rejected by day 38) (V). The graft was rejected immediately if the blood was derived from a haplotype-matched homozygous donor (VI). Prolongation of graft survival was also not achieved with fully mismatched DST (VII).

In pilot studies with purified CD4+ T cells from mice after haplotype-matched blood transfusion, Niimi et al. found that these cells had the ability to downregulate donor-specific CTL responses in a specific way. This finding indicates that allospecific Tregs had been induced by the blood transfusion and that these Tregs were involved in prolongation of graft survival.

According to Niimi et al., graft survival induced by the matched DST is probably due to priming of host T cells that recognize allopeptides presented by self(shared)-MHC indirectly on recipient APC and directly on donor APCs. However, since graft survival was also prolonged in combinations where the blood and organ donor shared only the mismatched haplotypes (IV and V), Niimi et al. suggest that presentation of allopeptides by recipient (self)-APCs is the essential pathway for tolerance induction.

Although indirect alloantigen recognition may have been essential to tolerance induction during the pretreatment regimen, the hypothetical-induced Tregs had to be reactivated by the transplanted organ for graft prolongation to occur. According to Niimi et al., this explains why the hearts in group III were all immediately rejected. In their own words: “… *the organ did not have a recipient haplotype and was therefore not able to present the allopeptide*.”

With regard to prolongation of graft survival in group II where the blood and organ donor share a recipient haplotype while being mismatched for the other, Niimi et al. suggest that the tolerance achieved is due to cross-reactivity or sharing of allopeptides between the mismatched haplotypes (haplotypes B and C).

Several mechanisms may have contributed to the prolongation of graft survival obtained in the Niimi et al. study. It is, for example, well known that alloreactive T cells anergize or are deleted when reacting with donor-derived allopeptides or allo-MHC complexes in non-inflammatory transplantation settings (57). Although such passive immunological conditions are unlikely to last long, they may create favorable immunological situations for the establishment of other more permanent and active regulatory mechanisms, such as the induction/conversion of Tregs. However, it can be questioned whether this “tolerogenic” immunological situation would have been crucial for the induction of tolerance in mice that were fully immune competent and never immunosuppressed.

The direct pathway of allorecognition is primarily associated with acute graft rejection (58). In experiments where tolerance is induced without the use of immunosuppressants, conventional effector T cells would be expected to be primed to reject the donor organ instead of being converted into Tregs. The hypothesis presented in this paper seeks an explanation for how Tregs in the Niimi experiment may be induced and cause prolongation of graft survival in an inflammatory setting.

### Donor Tregs and MHC-II Recruitment—Transfer of Self-tolerance to the Organ Recipient

All mice in the Niimi experiment were naive (i.e., immunologically inexperienced). Therefore, we also find it reasonable to assume that a substantial portion of the Tregs were self-specific tTregs, positively selected in the thymus upon encountering tissue-specific antigens before entering the periphery (51, 59). Because there is access to constant sources of self-antigens, self-specific tTregs may be in a more mature developmental stage than Tregs with allospecific TCRs (5). Based on these assumptions, we hypothesize that

I. Whole live blood contains functional self-specific Tregs, which are transferred to the recipient by blood transfusion. These self-specific donor-Tregs can be activated (if this is not already the case) and proliferate *via* the indirect pathway of antigen recognition/presentation, i.e., recipient APCs, if (a) the donor and recipient have a sufficient match of major histocompatibility complex (MHC) classes I and II, for example, share one haplotype and (b) the recipient APCs present the donor-derived peptide that is recognized naturally by the donor-derived Treg and the indirect antigen-presenting profile (MHC/peptide profile) is functionally identical to the immunological self of the blood donor, i.e., that at least some self-peptide/MHC complexes are identical in the recipient and blood donor, the antigenic background on which that these blood donor-derived Tregs have been selected on.

**Figure 2:**
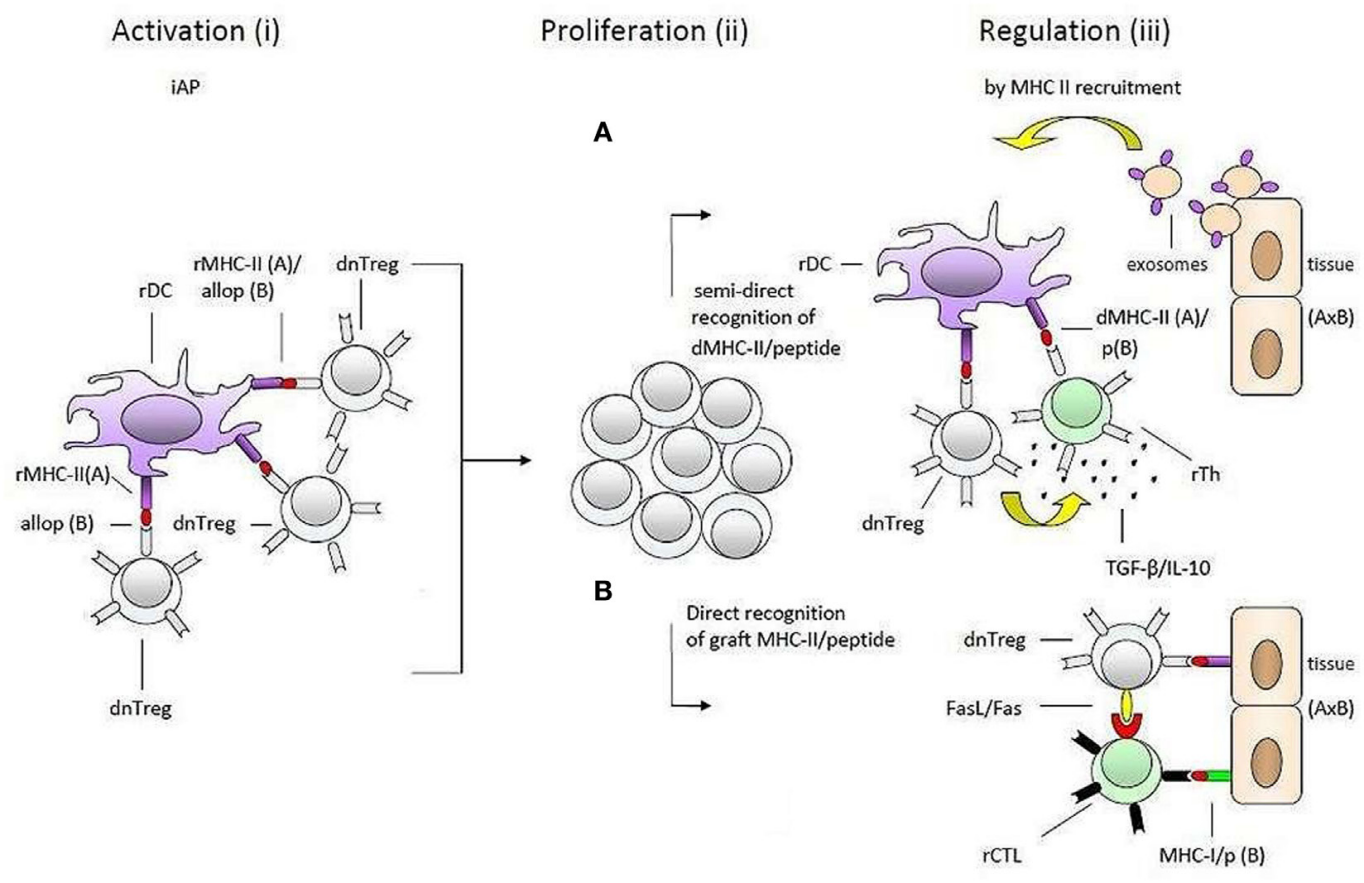
**Transplantation tolerance by donor tTregs and major histocompatibility complex class II (MHC-II) recruitment**. A simplified presentation of the hypothesis of one haplotype-matched donor-specific blood transfusion-induced allotolerance in the study by Niimi et al. The process is divided into two main phases: (1) what may happen in the time period between blood transfusion and transplantation (i) and (ii) and (2) after transplantation (iii). Donor-derived self-specific tTregs become (i) activated and (ii) proliferate by recognizing self-MHC-II/allopeptide complexes on host antigen-presenting cells that are functionally identical to the antigenic background they were selected on in thymus. During this preliminary phase of tolerance induction, activated donor tTregs may induce linked suppression and infectious tolerance before transplantation (not shown). These regulatory T cells (Tregs) may be reactivated after transplantation when encountering self-MHC-II/peptide complexes on the solid organ allograft (iii). According to the hypothesis, the enhancement of transplantation tolerance in non-immune-suppressed recipients is dependent on MHC-II recruitment (upregulation of MHC-II expression) by epithelial and endothelial cells within the solid organ allograft. As suggested by the hypothesis downregulation of the alloresponse may be effectuated by two different pathways: **(A)** recognition of intact donor organ MHC-II/peptide complexes which are taken up and presented by rDCs, e.g., *via* trogocytosis, shed MHC-II/peptide complexes (not shown) or fusion with exosomes, and **(B)** by binding directly to the allograft. In both cases (pathways A and B), donor-Tregs may physically out-compete naive alloreactive T cells, suppress or kill neighboring donor reactive effector T cells, or convert them into secondary regulatory cells (induce infectious tolerance). The regulatory effector mechanisms included in the figure, IL-10 and TGF-β in pathway A (pictured as small black dots) and FasL/Fas in pathway B, serve only as a few examples and are randomly placed. So are the recipient effector T cells. Further, the effector mechanisms may be operating together in each pathway. dnTreg, donor tTregs; dMHC, donor MHC; rMHC, recipient MHC; rTh, recipient Th; rDC, recipient DC.

As illustrated in Figure 2, our model suggests that self-specific donor tTregs recognize MHC-II/allopeptide complexes on host APCs that are functionally identical to the immunological self of the donor. There is a possibility that the transferred self-specific donor Tregs would be more easily activated by antigenic stimuli because they are positively selected in the thymus and are probably at a more advanced stage of development than naive allospecific recipient T cells. Additionally, *in vitro* studies have shown that Foxp3 Tregs can physically out-compete non-Tregs in aggregating around APCs (52).

This situation could delay or even impair the establishment of a response against antigens from the blood donor and allow large-scale multiplication of donor Tregs before the organ is transplanted.

As indicated by the different outcomes of allograft survival in group I and, i.e., II and IV, the hypothetical multiplied self-specific donor tTregs appear to be restricted to carrying out their full regulatory potential of responses against a genetic identical organ donor. We further hypothesize that

II. As a vital part of the maintenance of immunological self-tolerance, (a) responses directed against organs (either as a HVG-reaction or auto-reactive) cause the cells of the attacked organ (endothelial and epithelial cells) to produce or upregulate the production of MHC-II molecules. We have chosen to term the upregulation of MHC-II “MHC-II recruitment.” MHC-II recruitment allows for activated self-specific Tregs (and recipient Tregs with direct allospecificity) to bind specifically to the organ and downregulate immune responses at the site of inflammation. This self-protecting mechanism will be activated if or when the transplanted organ is attacked by the host immune system.

The specific acquired tolerance in recipients that were transfused with one haplotype-matched DST can be explained by MHC-II recruitment by the endothelial/epithelial cells of the transplanted organ. From here, two pathways, which are not mutually exclusive, can support Tregs in performing their memory function and maintaining allotolerance: (1) semi-direct recognition of MHC-II/peptide complexes transferred directly by epithelial cells to recipient DCs (rDCs) or by epithelial exosomes and membrane vesicles (60–62); (2) direct binding to cognate MHC-II/peptide complexes on the epithelial and endothelial cells. Both suggestions would give donor-Tregs the ability to suppress or kill bystander donor reactive effector T cells or even convert them into secondary regulatory cells (63). Conversion and suppression of effector T cells by activated donor tTregs may already occur *via* rDCs before the donor organ is transplanted. The hypothetical induction of pTregs with specificity for a different MHC-II/peptide combination (D/b) than that of the blood donor-derived tTregs can explain prolongation of graft survival in group IV.

The suggestion that MHC-II recruitment is essential for Tregs to achieve their full regulatory potential, both in transplantation tolerance and peripheral immunological self-tolerance, is consistent with the following discoveries: (1) several studies have demonstrated that endothelial and epithelial cells in human transplants and in mice upregulate their production of MHC-II molecules (64–66). (2) In a colitis-induced mouse model, a strong correlation was found between IFN-γ-induced production of MHC-II in intestinal epithelial cells and the protection of colitis (67). (3) It has recently been demonstrated that IFN-γ-induced production of MHC-II in vascular endothelium in mice promotes Treg trafficking to the site of inflammation by specific antigen recognition of the endothelium. Furthermore, the influx of Tregs in this study did eventually result in a significant decrease in the influx of effector T cells to the target tissue (68).

The attachment of Tregs to endothelial/epithelial MHC-II/peptide complexes may even be a short cut to reactivation of Tregs (memory Tregs) as suggested by Niimi et al. 17 years ago. Although activation of T lymphocytes is closely linked to professional APCs, recent studies have shown that hepatocytes from a transgenic mouse model, expressing stable MHC-II and featuring CD80, are able to activate CD4+ T cells. In this study, the epithelial activation of CD4+ T cells was not associated with autoimmune reactions (69). Additionally, it has recently been suggested by Kambayashi and Laufer that the production of MHC-II in non-hematopoietic cells may contribute to tolerance by stimulating Tregs (70). The latter may explain why spontaneous tolerance toward liver transplants is more frequent compared to other solid organ allografts (71).

Although our hypothesis is about transplantation tolerance induced by donor tTregs, recipients of one haplotype-matched allografts may, like the donor, harbor self-specific tTregs that recognize and bind self-MHC-II/peptide complexes on the donor organ. These hypothetical recipient tTregs may have been a contributing factor to prolongation of graft survival in recipients that were not tolerated for the mismatched antigen in the donor organ, as in group II. However, since tolerance was not achieved with matched pretransplanted homozygous donor blood (A in group VI), recipient and donor tTregs appear to be incapable of inducing tolerance without the presence of an alloantigen, for example, allopeptides from shared minor antigens between the blood donor (haplotype B) and the organ donor (haplotype C) as indicated by the outcome in group II (Figure 1).

## Summary and Future Perspectives

In this article, we have introduced a novel hypothesis on DST-induced transplantation tolerance based on a re-interpretation of a 17-year-old immunogenetic study by Niimi et al. (13). The animals in this study never received any immunosuppressive drug, which is unusual and excludes interference of artificially forced immune phenomena, such as antibody-induced clonal anergy. We find that such experiments facilitate the analysis and may allow other mechanisms to become more apparent.

Based on the immune status of the mice in the experiment of Niimi et al., two main suggestions have been presented in the hypothesis: (1) blood donor-derived self-specific tTregs can be (re)activated and multiplied by indirectly recognizing MHC/peptide complexes on host APC that correspond to the antigenic background they have been selected on in thymus; (2) the transplanted organ upregulates endothelial/epithelial expression of MHC-II if or when the organ is attacked by the host immune system. It is further suggested that the multiplied donor tTregs suppress bystander allospecific T cells and may induce infectious tolerance by binding directly to the donor organ or semi-directly to professional host APCs.

This hypothesis is consistent with recent animal studies which have shown that for long-term transplantation tolerance to occur, there is a need for Tregs that recognize allopeptides presented by self-MHC-II on host and donor APCs.

Several studies have indicated that tTregs constitute a more stable and mature cell population than Tregs induced from the naïve T-cell pool. Therefore, approaches that focuses on self-specific donor tTregs with specificity for self-peptides presented by shared MHC-II could be an attractive shortcut to prolongation of allograft survival because many apparently difficult steps in the generation of new Tregs from the naïve T cell pool may already have been completed. Yet, introduction of donor-specific antigens, such as donor blood and donor Tregs, prior to transplantation may increase the risk of immunizing the host.

According to our hypothesis, recipients of one haplotype-matched allografts may, just like the donor, harbor self-specific tTregs that can recognize and bind to both pathways of alloantigen presentation. Expansion of such recipient tTregs could minimize the risk of immunizing the host. This mechanism may have contributed the moderate prolongation of graft survival in group II. However, it is crucial to find out why tolerance was not induced with haplotype-matched homozygous donor blood (group VI).

To further support the hypothesis proposed in this manuscript, it will be important to test experimentally the longevity of Tregs alive in the recipient and whether these Tregs are essential for tolerance induction. It is also important to test whether the transplanted organ upregulates the production of MHC-II and whether these donor MHC II/peptide complexes are transferred to recipient APCs/DCs. Finally, support of the hypothesis will also require testing of whether linked suppression and infectious tolerance occurs in the donor:recipient combination.

## Author Contributions

We hereby confirm that all the authors’ contribution to this manuscript meets with the criteria of authorship demanded by Frontiers.

## Conflict of Interest Statement

The authors declare that the research was conducted in the absence of any commercial or financial relationships that could be construed as a potential conflict of interest.
